# d-allulose Ameliorates Metabolic Dysfunction in C57BL/KsJ-db/db Mice

**DOI:** 10.3390/molecules25163656

**Published:** 2020-08-11

**Authors:** Dayoun Lee, Youngji Han, Eun-Young Kwon, Myung-Sook Choi

**Affiliations:** 1 Department of Food Science and Nutrition, Kyungpook National University, 1370 San-Kyuk Dong Puk-Ku, Daegu 702-701, Korea; dayoon1746@naver.com (D.L.); youngji.kor.han@gmail.com (Y.H.); 2 Center for Food and Nutritional Genomics Research, Kyungpook National University, 1370 San-Kyuk Dong Puk-Ku, Daegu 702-701, Korea

**Keywords:** type 2 diabetes mellitus, d-allulose, sugar substitute, metabolic syndrome

## Abstract

d-allulose is an uncommon sugar that provides almost no calories when consumed. Its sweetness is 70% that of sucrose. d-allulose is a metabolic regulator of glucose and lipid metabolism. However, few reports concerning its effect on diabetes and related metabolic disturbances in db/db mice are available. In this study, we evaluated d-allulose’s effect on hyperglycemia, hyperinsulinemia, diabetes and inflammatory responses in C57BL/KsJ-db/db mice. Mice were divided into normal diet, erythritol supplemented (5% *w*/*w*), and d-allulose supplemented (5% *w*/*w*) groups. Blood glucose and plasma glucagon levels and homeostatic model assessment (HOMA-IR) were significantly lower in the d-allulose group than in the normal diet group, and plasma insulin level was significantly increased. Further, d-allulose supplement significantly increased hepatic glucokinase activity and decreased hepatic phosphoenolpyruvate carboxykinase and glucose-6-phosphatase activity. Expression of glucose transporter 4, insulin receptor substrate 1, phosphatidylinositol-4,5-bisphosphate 3-kinase catalytic subunit alpha and AKT serine/threonine kinase 2 were also upregulated by d-allulose supplement in adipocyte and muscle. Finally, d-allulose effectively lowered plasma and hepatic triglyceride and free fatty acid levels, and simultaneously reduced hepatic fatty acid oxidation and carnitine palmitoyl transferase activity. These changes are likely attributable to suppression of hepatic fatty acid synthase and glucose-6-phosphate dehydrogenase activity. Notably, d-allulose also reduced pro-inflammatory adipokine and cytokine levels in plasma. Our results indicate that d-allulose is an effective sugar substitute for improving lipid and glucose metabolism.

## 1. Introduction

Type 2 diabetes mellitus (T2DM) is a metabolic disease caused by genetic or environmental factors, or both, including insufficient or relative deficiency of insulin and dysfunction of pancreatic islets, leading to chronic hyperglycemia [1,2]. Free fatty acids (FFA) in plasma may be intermediaries in impaired insulin sensitivity and glucose tolerance related to obesity and T2DM [3,4,5]. Adipocytes display insulin resistance, indicating that FFA transport to the liver by the portal vein is increased when visceral triglyceride stores are elevated [6,7]. Hepatic function is essential for maintaining glucose homeostasis, and lipid accumulation in the liver may be an important factor for insulin resistance [8,9]; hepatic insulin resistance is associated with increased production of FFA. Circulating FFA levels are commonly elevated in obese and diabetic subjects, and increased FFA levels curb insulin suppression of hepatic glucose production by activating gluconeogenesis and inhibiting glycolysis [10,11,12]. Specifically, elevated fatty acid oxidation promotes excessive hepatic gluconeogenesis and suppresses glucose utilization [13]. A decrease in circulating FFA and fatty acid oxidation is expected to improve hyperglycemia and strengthen insulin response by suppressing glucose production and increasing glucose utilization [14].

Global prevalence of T2DM was estimated to be 9.3% in 2019 and is expected to increase to 10.2% by 2030. The condition is a leading worldwide risk factor for mortality [15]. Overconsumption of sugar is a primary dietary factor for producing T2DM. An alternative sweetener for sugar is therefore highly desirable [16]. d-allulose has low energy density, exhibiting almost zero calories, and is a metabolic regulator of glucose and fat metabolism in a number of studies [17,18,19]. However, studies on d-allulose on T2DM caused by genetic defects are lacking. The db/db mouse model of leptin deficiency is the most widely used mouse model of T2DM. These mice display a mutation in the gene encoding the leptin receptor, and leptin deficiency confers susceptibility to obesity, insulin resistance, and T2DM [20]. We hypothesized that d-allulose could counter this genetic defect and thus investigated the impact of d-allulose on diabetes and its related metabolic disorders in C57BL/KsJ-db/db mice.

## 2. Results

### 2.1. d-allulose Supplementation Reduced Body Weight and Plasma Lipid Content, and Regulated mRNA Expression of WAT

**Figure 1:**
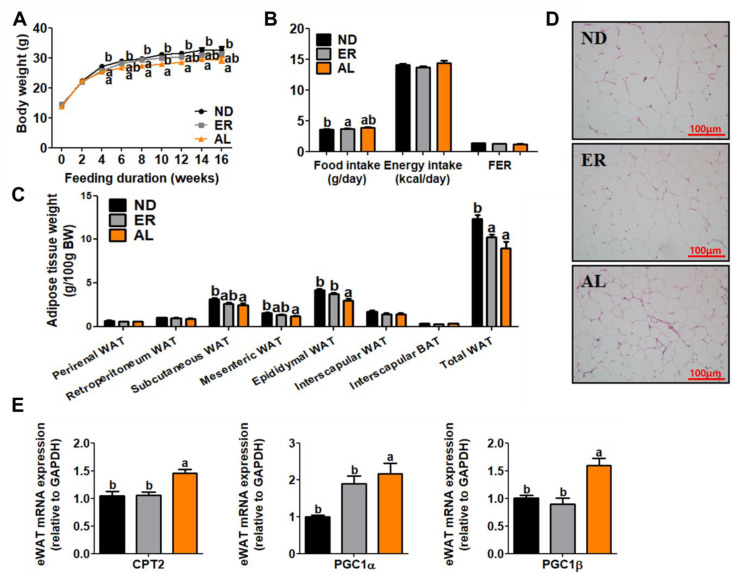
Effect of AL supplementation for 16 weeks on: body weight (**A**), food efficiency ratio (FER) (**B**), adipose tissue weight (**C**), epdidymal white adipose tissue morphology (200× magnification) (**D**) and mRNA expression (**E**). Data are mean ± S.E. ^a b^ Means values with unlike superscript letter are significantly different (*p* < 0.05); WAT, white adipose tissue; Representative photomicrographs of the liver are shown at 200× magnification. CPT2, carnitine palmitoyltransferase II; PGC, peroxisome proliferator-activated receptor gamma coactivator.

Dietary supplementation with d-allulose significantly decreased body weight compared with ND group mice without altering food and energy intake or food efficiency ratio (FER) (Figure 1A,B). Also, epididymal white adipose tissue (WAT) size and weight of subcutaneous WAT, mesenteric WAT, epididymal WAT and total WAT were significantly decreased in the AL group compared with controls (Figure 1C,D). Moreover, d-allulose significantly suppressed expression of *UCP2*, *PGC1α* and *PGC1β* genes related to fatty acid oxidation (Figure 1E). Plasma triglyceride (TG) and free fatty acid (FFA) levels after 16 weeks of feeding were significantly decreased in the AL group compared to controls (Table 1). No statistical differences in plasma total cholesterol (TC) or non-high density lipoprotein cholesterol (nonHDL-C) levels were observed between ER and AL groups compared to control mice. AL supplementation tended to increase apolipoprotein (Apo) A-I level and decrease Apo B level compared to ND feeding, and the Apo A-I/Apo B ratio was increased in the AL group.

### 2.2. d-allulose Supplementation Amended Insulin Resistance through the Regulation of Enzyme Activities Related to Glucose Metabolism.

**Figure 2:**
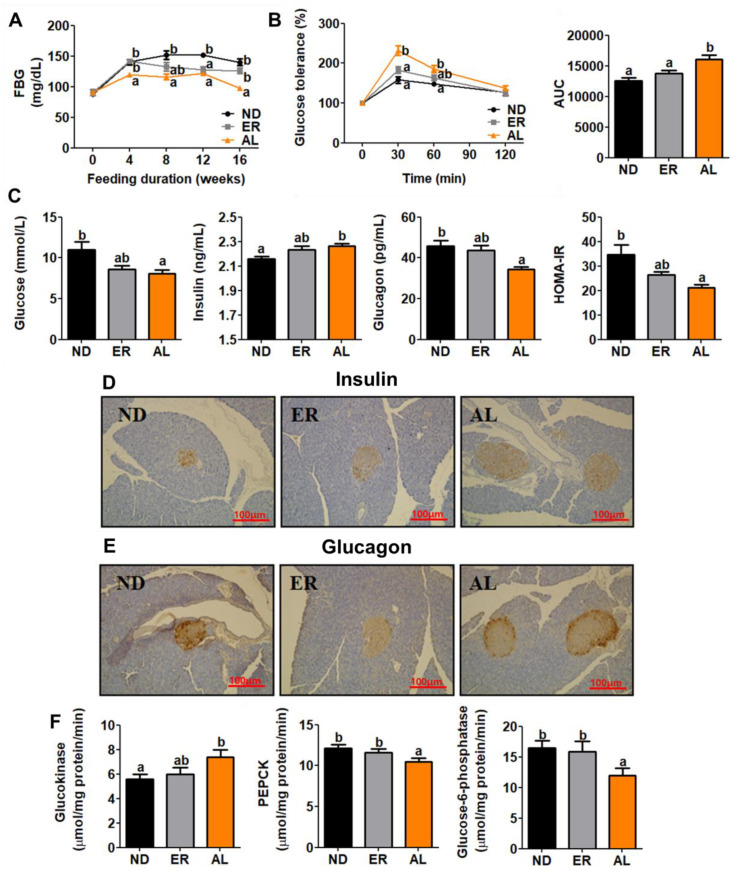
Effect of AL supplementation for 16 weeks on; FBG (**A**), IPGTT (**B**), plasma glucose, insulin and glucagon, and HOMA-IR (**C**), pancreatic immunohistochemistry analysis (200X magnification) (**D**,**E**), and enzyme activities related glucose metabolism (**F**). Data are mean ± S.E. ^a b^ Means values with unlike superscript letter are significantly different (*p* < 0.05); FBG, fasting blood glucose; AUC, area under the curve; PEPCK; phosphoenolpyruvate carboxykinase.

Fasting blood glucose (FBG) in AL group mice was significantly lower in controls beginning in the 4th week of feeding (Figure 2A). Intraperitoneal glucose tolerance test (IPGTT) and area under the curve (AUC) results indicated that d-allulose ameliorated glucose intolerance (Figure 2B). Also, AL group mice displayed significantly decreased plasma glucose and glucagon levels and HOMA-IR, along with increased insulin levels (Figure 2C). Immunohistochemistry staining analysis for pancreatic insulin and glucagon were consistent with these results and showed that islet cells were well preserved in AL supplemented animals (Figure 2D). Moreover, d-allulose consumption decreased phosphoenolpyruvate carboxykinase (PEPCK) and Glucose-6-phosphatase (G6P) activity involved in gluconeogenesis, and significantly increased glucokinase (GK) activity involved in glycolysis compared with ND fed mice (Figure 2F).

### 2.3. d-allulose Increased mRNA Expression of Genes Related Glucose-Metabolism

**Figure 3:**
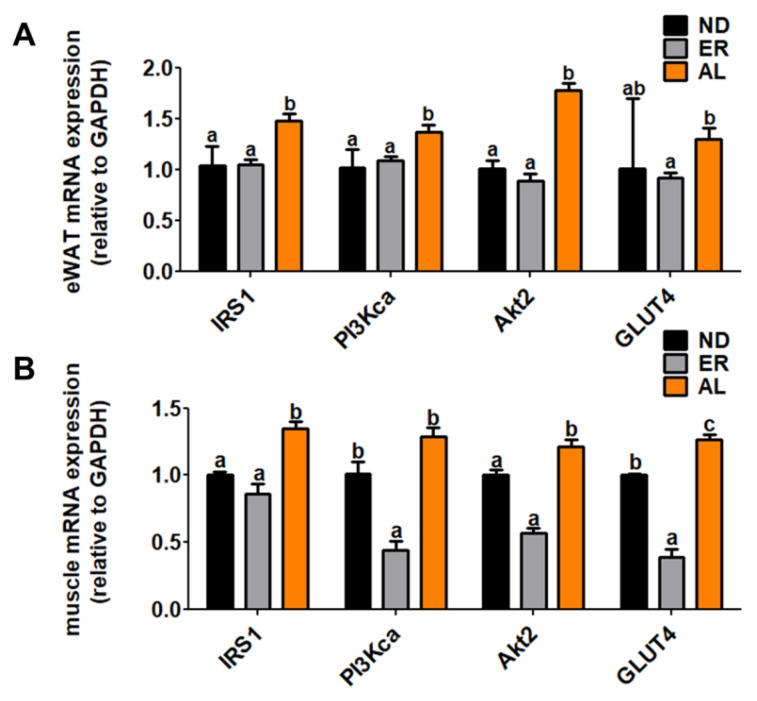
Effect of AL supplementation for 16 weeks on (**A**) WAT and (**B**) myocyte glucose metabolism-related gene expression in C57BL/KsJ-db/db mice. Data are mean ± S.E. ^a b^ Means values with unlike superscript letter are significantly different (*p* < 0.05); IRS1, insulin receptor substrate 1; PI3Kca, phosphatidylinositol-4,5-bisphosphate 3-kinase catalytic subunit alpha; Akt2, AKT serine/threonine kinase 2; GLUT4, facilitated glucose transporter 4.

The dietary d-allulose significantly increased expression of *IRS1*, *PI3Kca* and *AKT2* mRNA in epididymal WAT compared with other groups (Figure 3). Likewise, mRNA expression of *IRS1* and *Akt2* in muscle tissue was markedly increased.

### 2.4. d-allulose Supplement Reversed Hepatic Steatosis

**Figure 4:**
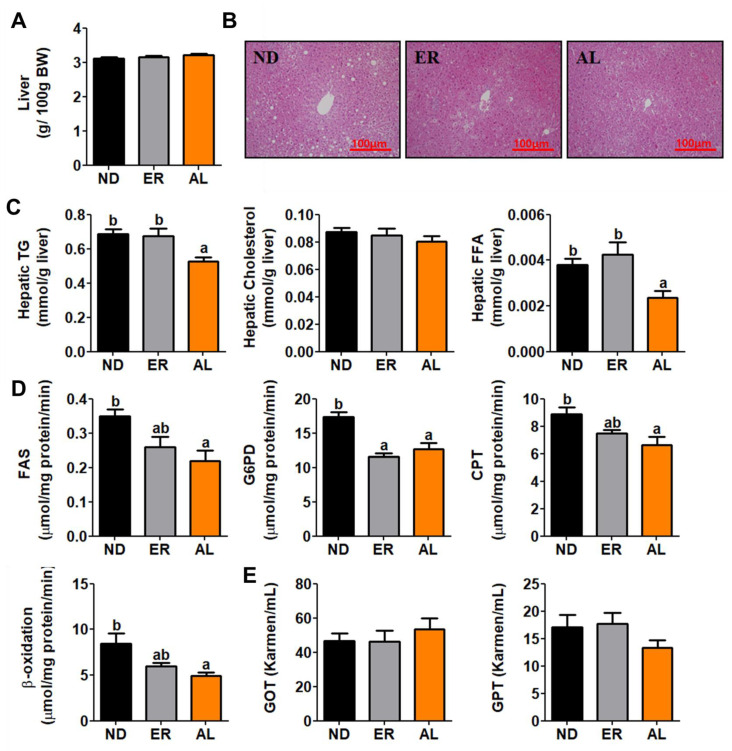
Effect of AL supplementation for 16 weeks on; liver weight (**A**), hepatic morphology (**B**), hepatic lipid contents (**C**), lipid-metabolism regulating enzyme activities (**D**), and hepatoxicity biomarkers (**E**). Data are mean ± S.E. ^a b^ Means values with unlike superscript letter are significantly different (*p* < 0.05); Hematoxylin and eosin (H&E) stained transverse section of liver; Representative photomicrographs of the liver are shown at 200× magnification; TG, triglyceride; FAS, fatty acid synthase; G6PD, glucose-6-phostate dehydrogenase; CPT, Carnitine palmitoyltransferase; GOT, glutamic oxaloacetic transaminase; GPT, glutamic pyruvic transaminase.

No significant difference in liver weight were found among treatment groups (Figure 4A), hematoxylin and eosin (H&E) staining analysis revealed that d-allulose suppressed hepatic lipid accumulation, and hepatic lipid contents in AL group mice was significantly decreased compared with controls (Figure 4B,C). Activities of fatty acid synthase (FAS), glucose-6-phosphate dehydrogenase (G6PD), carnitine palmitoyl transferase (CPT) and β-oxidation enzymes that regulate hepatic lipid metabolism were also significantly lower in the AL group (Figure 4D). Hepatotoxicity was indicated by plasma levels of glutamic oxaloacetic transaminase (GOT) and glutamic pyruvic transaminase (GPT) (Figure 4E); however, these levels were not significantly different among treatment groups.

### 2.5. d-allulose Supplementation Decreased Adipokine/Cytokine Levels

**Figure 5:**
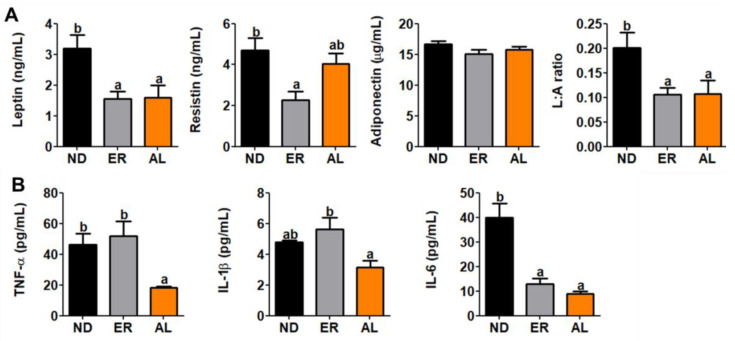
Effect of AL supplementation for 16 weeks on plasma (**A**) adipokine/(**B**) cytokine levels in C57BL/KsJ-db/db mice. Data are mean ± S.E. ^a b^ Means values with unlike superscript letter are significantly different (*p* < 0.05); TNF-α, tumor necrosis factor-alpha, IL, interluekin.

Plasma leptin levels and leptin:adiponectin (L:A) ratios were significantly decreased in AL group mice compared with controls (Figure 5A). Similarly, plasma inflammatory cytokine levels, TNF-α, IL-1β and IL6, were significantly decreased in the AL group (Figure 5B).

## 3. Discussion

The present study examined the ability of d-allulose to regulate glucose and lipid metabolism in db/db mice, a T2DM model, along with its impact on overall metabolic regulation.

C57BL/KsJ-db/db mice, characterized by obesity, infertility, hyperphagia, temporary hyperinsulinemia, hyperlipidemia, and hyperglycemia due to a leptin receptor mutation, were selected as an appropriate model for early-stage T2DM; these mice exhibit hepatic insulin resistance [21]. Many studies indicate that d-allulose can reduce body weight in diet-induced obese and db/db mice [18,22,23]. Our previous research showed that d-allulose normalizes body weight in HFD-induced obesity through regulation of enzyme activity and mRNA expression in the small intestine, liver, and epididymal WAT [18]. The current study is consistent with these findings and shows that dietary d-allulose supplementation significantly decreases body weight and body fat weight, without significant influence on FER. Also, morphological observations of lipid droplet in epididymal WAT suggested that ND group mice displayed larger epididymal WAT adipocytes than AL group animals. Further, mRNA expression of genes related to fatty acid oxidation was significantly upregulated in the AL group compared with controls.

Metabolic dysregulation of FFA is closely associated with insulin resistance and T2DM [24]. Preferential oxidation of FFA over glucose plays a major role in insulin sensitivity and metabolic disturbances of T2DM, as suggested by the glucose-fatty acid cycle of Randle [25]. Inflammation and oxidative stress are also important processes that can lead to insulin resistance [26]. Modulation of transcription by FFA via binding to peroxisome proliferator-activated receptors could eventually contribute to impaired glucose metabolism [27]. An increase in free fatty acid flux, resulting from increased lipolysis secondary to adipose-tissue insulin resistance, induces or aggravates insulin resistance in liver and muscle. This resistance is caused directly or indirectly, from triglyceride deposits, by generation of metabolites that alter insulin signaling [28]. Alleviating excess FFA is a target for treatment of insulin resistance. In this study, d-allulose markedly decreased plasma and hepatic free fatty acid content, significantly reduced hepatic FAS activity, and markedly increased mRNA expression of genes related to fatty acid oxidation in epididymal WAT.

Insulin signaling in adipose tissue and muscle is initiated by insulin binding to its receptor on cell membranes [29]. The PI3K-AKT signaling pathway plays an important role in the control of insulin metabolism in obesity and T2DM [30]. IRS1 activates PI3K by binding to its SH2 domain and activated PI3K stimulates AKT. Eventually, these signaling pathways result in the translocation of GLUT4 to the plasma membrane, leading to an increase in adipocyte glucose uptake [31]. Expression of IRS1, PI3Kca and AKT2 were significantly upregulated by AL supplementation that also tended to increase GLUT4 mRNA expression. IRS1, AKT2 and GLUT4 expression in muscle were markedly upregulated in AL group mice. We previously analyzed hepatic transcription patterns and biological pathways associated with d-allulose responsive genes. d-allulose ameliorated dysglycemia in diet-induced obese mice and significantly decreased mRNA expression related to PI3K-AKT signaling [32]. The current study suggests that regulation of gene expression and enzyme activity in mice fed with AL supplementation might lead to decreases in fasting blood glucose. Fasting blood glucose concentrations during the experimental period were decreased in AL group mice. Plasma glucose, insulin, and glucagon and HOMA-IR levels were measured to assess the effects of AL supplementation on insulin resistance. A lower HOMA-IR value is expected to reflect higher insulin sensitivity; higher HOMA-IR values lower insulin sensitivity or insulin resistance [24]. Our results indicate increased insulin levels after AL supplementation compared to controls. Further, this result is consistent with immunohistochemistry staining for pancreatic insulin. Chronic insulin deficiency and impaired insulin sensitivity are major causes of decreased glucose utilization and increased glucose production in several animal models of T2DM. Insulin decreases hepatic glucose output by activating glycogen synthesis and glycolysis, and by inhibiting gluconeogenesis [33]. In the present study, AL fed mice showed drastically increased hepatic GK activity, and decreased hepatic PEPCK and G6Pase activities in liver tissue. d-allulose also regulated hepatic enzyme activity related glucose-metabolism in high fat diet-induced T2DM mice model [32].

Insulin resistance is a metabolic dysfunction often mediated by increased inflammation [34]. Adipokines are a large group of pro-inflammatory mediators, including TNF-α, IL-6 and IL-1β. Expression of TNF-α and IL-6 are increased in obesity and insulin resistance [34,35,36,37]. Hyperglycemia results in a high level of IL-6, and treatment with IL-6 induces hyperglycemia and insulin resistance [35]. IL-1β is a central factor affecting TG accumulation [37]. Further, IL-1β effects expression of the lipogenic enzyme, FAS, in primary hepatocytes [38]. We measured pro-inflammatory cytokine concentrations of TNF-α, IL-1β and IL-6. Plasma TNF-α and IL-6 levels in AL group mice were significantly decreased, and levels of IL-1β tended to decrease compared to controls. Leptin concentration in plasma is increased in obesity, and high levels of leptin lead to the production of pro-inflammatory cytokines, including TNF-α and IL-6 [39,40,41]. Reduced plasma leptin level in the AL group may affect levels of TNF-α and IL-6 in plasma and reflect changes in body weight and body fat weight. Resistin is also a secreted adipokine, and promotes inflammation and insulin resistance [42]. AL supplement tended to decrease plasma resistin levels compared to controls [43]. d-allulose in a high-fat diet reduced diet-induced obesity and markedly decreased plasma inflammatory cytokine levels and related mRNA expression [44]. These findings show that AL supplementation can reduce inflammatory responses that may contribute to reduced weight gain and T2DM in db/db mice. In accordance with our results, citrus unshiu peel (CPE) is a rich source of citrus flavonoids that possess anti-inflammatory, anti-diabetic and lipid-lowering effects. In male C57BL/KsJ-db/db mice fed a normal diet with 2% CPE (2 g/100 g diet) for 6 weeks, mice supplemented with the CPE showed a significant decrease in body weight gain, body fat mass and blood glucose level. The anti-hyperglycemic effect of CPE appeared to be partially mediated through the inhibition of hepatic gluconeogenic PEPCK mRNA expression and its activity and through the induction of insulin/glucagon secretion. CPE also ameliorated hepatic steatosis and hypertriglyceridemia via the inhibition of gene expression and activities of the lipogenic enzymes and the activation of fatty acid oxidation in the liver. These beneficial effects of CPE may be related to increased levels of anti-inflammatory adiponectin and IL-10, and decreased levels of pro-inflammatory markers (IL-6, monocytechemotactic protein-1, interferon-γ and TNF-α) in the plasma or liver.

The limitations of this study should be emphasized. As a Supplementary Table S1, the ND group has 3.902 kcal/g and ERY and ALL groups have 3.702 kcal/g because erythritol and d-allulose has no calories. Thus, it is still unclear whether d-allulose can improve the metabolic status in db/db mice without having an impact of energy density. Accordingly, another study is underway, in which we will confirm the hypoglycemic effects of d-allulose in iso caloric pair-fed manner.

## 4. Materials and Methods

### 4.1. Animals and Diets

All procedures involving animals were approved by the Ethics Committee for animal studies at Kyungpook National University, Republic of Korea (Approval No. KNU 2017-93). Male C57BL/KsJ-db/db (4-wk old, n = 36) were purchased from Jackson Laboratory (Bar Harbor, ME, USA). We selected male mice as experimental animals to avoid positive metabolic control by female hormones in diabetes [45]. All mice were maintained in a room with controlled temperature (20–23 °C) and lighting (alternating 12 h periods of light and dark) and fed a pelletized commercial non-purified diet for one week after arrival. Mice were then randomly divided into three groups (n = 12) and fed experimental diets for 16 weeks (Supplementary Table S1) All mice were fed normal control diet (American Institute of Nutrition AIN-76 semisynthetic diet). The control group (ND) received diet without supplementation. Experimental groups received normal diet supplemented with either erythritol (ER, 5% (*w*/*w*) substituted for sucrose in normal diet) or d-allulose (AL, 5% (*w*/*w*) substituted for sucrose in normal diet). Erythritol was purchased from sigma Aldrich (Saint Louis, MO, USA) and d-allulose was supplied by CJ CheilJedang Corp. (Seoul, Korea).

### 4.2. Lipid Profile Analysis

Plasma and hepatic lipid concentrations were determined using commercially available kits. Plasma TG, TC and high-density lipoprotein cholesterol (HDL-C) levels were determined using Asan enzymatic kits (Asan, Seoul, Korea). Plasma ApoA-I and Apo B were measured using enzymatic kits (Eiken, Japan). Plasma FFA was measured using Nittobo enzymatic kits (Nittobo Medical Co., Tokyo, Japan).

Hepatic lipid was extracted as previously described [11], and dried lipid residues were dissolved in 1 mL of ethanol for TG, TC and FFA assays. Triton X-100 and sodium cholate in distilled water were added to 200 μL of dissolved lipid for emulsification. TG, TC and FFA concentrations were measured with the same enzymatic kits used for plasma analysis.

### 4.3. Blood Glucose Analysis

Blood glucose levels were measured with a glucose analyzer (Asan Pharm Co., Seoul, Korea). Concentrations were recorded every four weeks in whole blood obtained from tail veins after a 12 h fasting period. Plasma insulin and glucagon were measured with enzyme-linked immunosorbent assay (ELISA) kits (R&D systems^TM^, a bio-techne brand, Minneapolis, MN, USA). Homeostatic index of insulin resistance (HOMA-IR) was calculated based on homeostasis assessment as: HOMA-IR = [fasting glucose (mmol/L) × fasting insulin (μL U/mL)]/22.5.

### 4.4. Plasma Adipokines and Cytokines

Plasma adipokines (leptin, resistin and adiponectin) and cytokines (TNF-α, IL-6 and IL-1β) were determined via a multiplex detection kit (MERCK, Kenilworth, NJ, USA).

### 4.5. Hepatic Enzyme Activity

FAS activity was measured spectrophotometrically as previously described (Nepokroeff et al. [13]). G6PD activity as described in Pitkanen et al. [14] CPT activity following the method of Markwell et al. [15]. Fatty acid β-oxidation was measured spectrophotometrically by monitoring the reduction of NAD to NADH in the presence of palmitoyl-CoA as described by Lazarow [16], with slight modification.

### 4.6. Glucose-Regulating Enzyme Activity

Liver was separated to cytosolic and microsomal fractions as previously described. GK activity was determined using a continuous spectrophotometric assay, as described by Newgard et al. [17], with slight modification. PEPCK activity was monitored in the direction of oxaloacetate synthesis using the spectrophotometric assay developed by Bentle and Lardy, with slight modification [18]. Microsomal fractions were used for the measurement of glucose-6-phosphatase (G6P).

### 4.7. Real-Time qPCR Analysis

Epididymal WAT, liver and muscle samples from each group of mice were prepared and analyzed as previously described [46]. Total RNA (1 μg) was reverse transcribed using a commercial kit (Qiagen, Germany). mRNA expression was quantified by real-time quantitative PCR, using a QuantiTects SYBR green PCR kit (Qiagen, Hilden, Germany) with a CFX96TM real-time PCR system (Bio-Rad, UK). Primers were used for detecting gene expression in liver and epididymal WAT (Supplementary Table S2). Amplification was performed as: 10 min at 90 °C, 15 s at 95 °C and 60 s at 60 °C for a total of 35 cycles. The cycle threshold values were normalized using GAPDH. Relative gene expression was calculated with the 2^−ΔΔ*CT*^ method [47].

### 4.8. Histological Analysis and Immunohistochemistry

Liver, epididymal WAT and pancreas were removed from mice and fixed in a buffer solution of 10% formalin. Fixed tissues were processed routinely for paraffin embedding, and 4 μm sections were prepared and stained with hematoxylin and eosin (H&E). For immunohistochemistry, islets were sectioned, fixed in hydrogen peroxide and washed in citrate buffer (pH 6.0). These sections were treated with blocking reagent (Ultra Tech HRP, Beckman Coulter, Brea, CA, USA) to prevent nonspecific binding and incubated with monoclonal antibodies against insulin and glucagon (SantaCruz Biotech. Inc, Dallas, TX, USA). Antibody reactivity was detected using HRP-conjugated biotin-streptavidin complexes and developed with diaminobensidine tetrahydrochloride as the substrate. Stained areas were imaged with an optical microscope with a magnifying power of ×200.

### 4.9. Statistical Analysis

Data were expressed as mean ± standard error of the mean (SEM). All statistical analyses were performed using SPSS (SPSS, Inc., Chicago, IL, USA). Significant differences among groups were identified by Tukey’s multiple range test, multiple comparison procedure with *p* < 0.05 as the threshold for significance.

## 5. Conclusions

d-allulose supplementation suppresses lipid accumulation by promoting lipolysis and alleviates a variety of metabolic disorders, including dyslipidemia, hyperglycemia and inflammation in db/db mice.

## Figures and Tables

**Table 1:** Effect of AL supplementation for 16 weeks on plasma lipid profiles in C57BL/KsJ-db/db mice fed normal diet.

	ND	ER	AL
TG (mmol/L)	1.84 ± 0.08 ^b^	1.86 ± 0.08 ^b^	1.44 ± 0.05 ^a^
TC (mmol/L)	3.12 ± 0.1	3.15 ± 0.09	3.05 ± 0.12
FFA (mmol/L)	0.91 ± 0.03 ^b^	0.98 ± 0.03 ^b^	0.76 ± 0.04 ^a^
HDL-C (mmol/L)	0.95 ± 0.04 ^b^	0.75 ± 0.07 ^a^	0.83 ± 0.09 ^a,b^
nonHDL-C (mmol/L)	2.19 ± 0.10	2.40 ± 0.07	2.22 ± 0.09
Apo A- I (mg/dL)	63.57 ± 1.85 ^b^	54.77 ± 1.85 ^a^	70.59 ± 1.99 ^c^
Apo B (mg/dL)	9.29 ± 0.22 ^b^	8.57 ± 0.56 ^ab^	7.72 ± 0.35 ^a^
Apo A- I/Apo B	7.07 ± 0.26 ^a^	6.81 ± 0.69 ^a^	9.32 ± 0.42 ^b^

Data are mean ± S.E. ^a,b,c^ Means values with unlike superscript letter are significantly different (*p* < 0.05); TG, triglyceride; TC, total cholesterol; FFA, free fatty acid; HDL-C, high density lipoprotein-cholesterol; nonHDL-C, non-high density lipoprotein-cholesterol; Apo A- I, apolipoprotein A-I; Apo B, apolipoprotein B.

## Supplementary Materials

The following are available online, Table S1: Composition of experimental diets (% of diet, *w*/*w*); Table S2: Primer sequences used for RT-qPCR validation of microarray data.
