# Supplementary material for: d-allulose Ameliorates Metabolic Dysfunction in C57BL/KsJ-db/db Mice
Source: Molecules. 2020 Aug 11;25(16):3656. doi: 10.3390/molecules25163656 (PMC7465492; doi:10.3390/molecules25163656)
Supplement: Supplementary file 1 [file molecules-25-03656-s001.pdf]

Supplementary Table S1. Composition of experimental diets (% of diet, w/w)

Ingredient(g)	ND	ER	AL
Casein	200.00	200.00	200.00
D,L-methionine	3.00	3.00	3.00
Corn starch	150.00	150.00	150.00
Sucrose	499.99	449.99	449.99
Cellulose powder	50.00	50.00	50.00
Corn oil	50.00	50.00	50.00
Mineral mixture (AIN-76) [1]	35.00	35.00	35.00
Vitamin mix (AIN-76) [2]	10.00	10.00	10.00
Choline bitartrate	2.00	2.00	2.00
tert-Butylhydroquinone	0.01	0.01	0.01
Erythritol	-	50.00	-
Allulose	-	-	50.00
Total(g)	1000.00	1000.00	1000.00
Calorie(kcal/g)	3.902	3.702	3.702

[1] AIN-76 mineral mixture (Harlan Teklad Co., Madison, WI, USA). [2] AIN-76 vitamin mixture (Harlan Teklad Co., Madison, WI, USA). ND: normal diet; ER: erythritol; AL: allulose.

Supplementary Table S2. Primer sequences used for RT-qPCR validation of microarray data

Gene	Primer Direction	Sequence
IRS1	Forward	5′-GGTACCTACAAGTGGGTGAA-3′
	Reverse	5′-AGATGGTGAAAAGGGAAGAT-3′
PI3Kca	Forward	5′-GGGTTTTCTGTCCCCTCTGA-3′
	Reverse	5′-TGATGTCTGGGTTCTCCCAA-3′
Akt2	Forward	5′-ACGTGGTGAATACATCAAGACC-3′
	Reverse	5′-GCTACAGAGAAATTGTTCAGGGG-3′
GLUT4	Forward	5′-CTGAGAACTTAACTGCTGAAG-3′
	Reverse	5′-AGGAGTTTGTTGGTGTATTTAC-3′
CPT2	Forward	5′-GCCTGCTGTTGCGTGACTG-3′
	Reverse	5′-TGGTGGGTACGATGCTGTGC-3′
PGC-1α	Forward	5′-AAGTGTGGAACTCTCTGGAACTG-3′
	Reverse	5′-GGGTTATCTTGGTTGGCTTTATG-3′
PGC-1β	Forward	5′-GGTCCCTGGCTGACATCCAC-3′
	Reverse	5′-GGCACATCGAGGGCAGAG-3′
GAPDH	Forward	5′-ACAATGAATACGGCTACAGCAACA-3′
	Reverse	5′-GGTGGTCCAGGGTTTCTTACTCC-3′

IRS1, insulin receptor substrate 1; PI3Kca, phosphatidylinositol-4,5-bisphosphate 3-kinase catalytic subunit alpha; Akt2, AKT serine/threonine kinase 2; GLUT4, facilitated glucose transporter 4; CPT2, carnitine palmitoyl-CoA carboxylase 2; PGC-1α and PGC-1β, peroxisome proliferator-activated receptor gamma coactivator 1-alpha and 1-beta; GAPDH, glyceraldehyde-3-phosphate dehydrogenase.

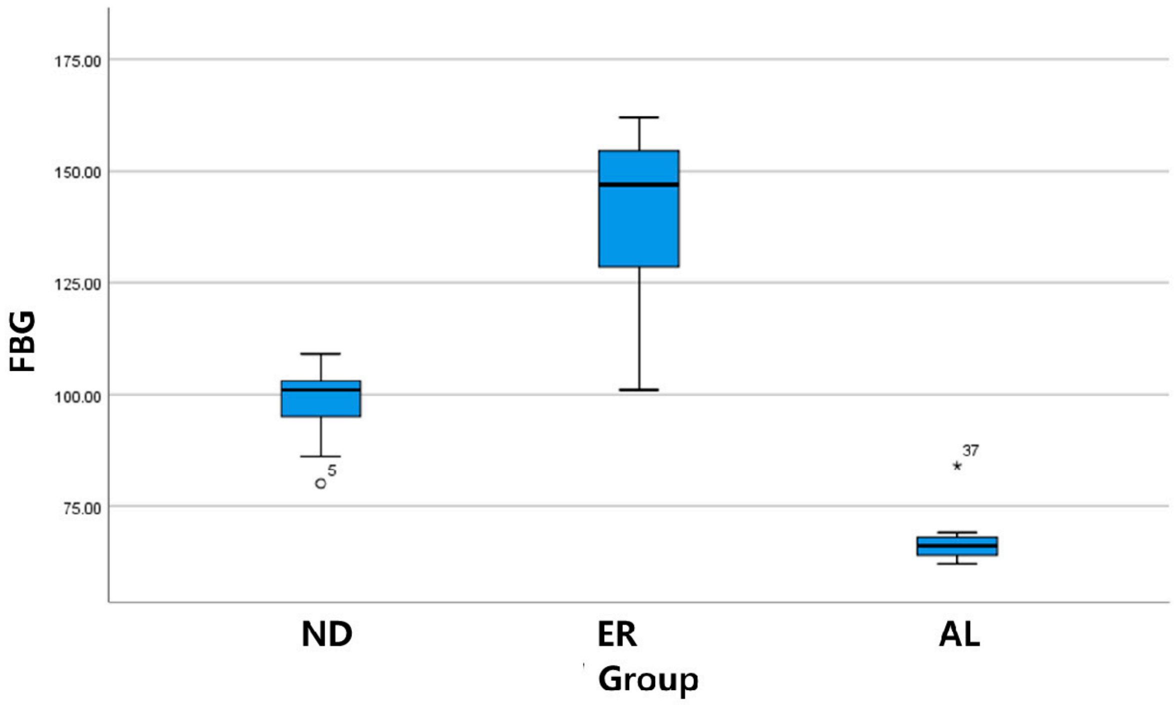

Supplementary figure S1
